# Lytic bacteriophages of *Gordonia rubripertincta* from topsoil in Lubbock, Texas: FlyingTortilla and ScarletRaider

**DOI:** 10.1128/mra.01333-24

**Published:** 2025-04-15

**Authors:** Laurissa N. Miller, Natalie Block, Whitney Dickens, Carson Bellew, Christian Deluna, Francesca Makilan, Malli Bhakta, Ashleigh Crawford, Trinity Criner, Chase Drucker, Aqsa Fayyaz, Jasmine Goh, Caitlyn Guetersloh, Claire Jansen, Dana Pham, Andrea Resendez, Austen Rowell, Fahareen B. Mosharraf, Allie C. Smith, Lisa M. Bono

**Affiliations:** 1 Department of Biological Sciences, Texas Tech University 6177, Lubbock, Texas, USA; 2 Teaching, Learning, and Professional Development Center, University Library, Texas Tech University, Lubbock, Texas, USA; 3 TrUE Scholars Program, Center for Transformative Undergraduate Experiences, Texas Tech University 6177, Lubbock, Texas, USA; 4 Honors College, Texas Tech University, Lubbock, Texas, USA; Portland State University, Portland, Oregon, USA

**Keywords:** SEA-PHAGES, bacteriophages, *Caudoviricetes*, viral genomics, sequence analysis, enriched isolation, gene prediction

## Abstract

We isolated two environmental phages, as part of the Science Education Alliance-Phage Hunters Advancing Genomics and Evolutionary Sciences program, that infect *Gordonia rubripertincta* from topsoil in Lubbock, Texas. We report the complete genome sequences of lytic bacteriophages FlyingTortilla and ScarletRaider. Sequence similarity analysis reveals the viruses as a part of an unclassified order within the *Caudoviricetes* class.

## ANNOUNCEMENT

*Gordonia rubripertincta* is a gram-positive bacterium found commonly in soil. *Gordonia* spp. have been of special interest as they are ecologically diverse and can degrade environmental pollutants (1). As part of the Science Education Alliance-Phage Hunters Advancing Genomics and Evolutionary Sciences discovery-based program, we isolated environmental bacteriophage from soil collected in Lubbock, Texas, that infects this bacterium (2).

The bacteriophages FlyingTortilla and ScarletRaider were collected on 30 August 2023. Topsoil was collected at the coordinates (33.56764, −101.89808) and (33.590509497217205, −101.86574082352972) for FlyingTortilla and ScarletRaider, respectively. The samples for each phage were suspended in peptone yeast calcium broth, centrifuged at 2,000 × *g* (3,500 rpm) for 10 minutes, and filtered using 0.22 µm syringe filters to remove debris and bacteria. The filtrate was then inoculated with 500 µL of *Gordonia rubripertincta* NRRL B-16540 culture, loosely capped, incubated at 30℃ at 220 rpm for 3 days, and then plated using an agar overlay technique (3). The resulting clear plaques from enrichments are consistent with a lytic lifestyle. These plaques were cut from the agar overlay plates and triple-plaque purified (3). Plaques were picked, placed into cryovials with 40% glycerol solution, vortexed, and then used to make lacy (webbed) plates to generate a high-titer lysate. The DNA of each sample was extracted from the lysate using the Quantabio Extracta Plus DNA kit. A BioTek Take3 plate was used to evaluate the concentration and quantity of the DNA. NEB Ultra II FS kits (v.3 reagents) were used to prepare the samples for sequencing on an Illumina MiSeq system to generate 150 bp single-end reads.

The reads were assembled using Newbler v2.9. Consed v29 was used for quality control of the assemblies and to identify each genome’s termini (4, 5). The GC content of FlyingTortilla is 60.3% with a total genome size of 92,983 base pairs and 125 predicted genes, while ScarletRaider has a 60.4% GC content with a total genome size of 92,813 base pairs and 127 predicted genes (Table 1). Genes and their positions were predicted using Glimmer v3.0 (6) and GeneMark v2.5 (7) and were manually validated using DNA Master v5.23.3 (8). Further annotation was completed using the Phage Evidence Collection and Annotation Network (9). Gene functions were predicted using a combination of BLAST v2.11.01 (using the Actinobacteriophage and NCBI nonredundant database), Phamerator Actino_Draft v402 (using Actino_draft database v578) (10–14), HHpred v3.0 (using the PDB_mmCIF70), Pfam v36, and NCBI Conserved Domain Database v3. All software was used with default preferences.

**TABLE 1:** Characterization of the reported bacteriophages

Parameter	Data for the isolated bacteriophages	
	FlyingTortilla	ScarletRaider
Genome length (bp)	92,983	92,813
Total number of reads	399,512	540,522
Sequencing coverage (×)	543	731
No. of predicted genes	125	127
No. of tRNAs	0	0
No. of hypothetical proteins of unknown function	86	90
GC content (%)	60.3%	60.4%
Facility performing sequencing	Pittsburgh Bacteriophage Institute	Pittsburgh Bacteriophage Institute
GenBank accession no.	PQ184803	PQ184837
SRA accession no.	SRR31899099	SRR31899096
BioProject accession no.	PRJNA488469	

FlyingTortilla and ScarletRaider were assigned to the DQ cluster based on gene content with a minimum cutoff of 35% similarity to phages in the Actinobacteriophage database, PhagesDB (https://phagesdb.org), and the genome termini were identified as circularly permuted (14). All members of DQ infect *Gordonia* spp. and have an average genome length of 89,667 bp with 124 genes. Although eight out of 18 members of this cluster encode for a single tRNA, no tRNAs were detected in ScarletRaider and FlyingTortilla using tRNAscan-SE v2.0 and ARAGORN v1.2.38 (15, 16), consistent with the rest of the members of the DQ cluster.

## Data Availability

This project has been deposited in GenBank under accession numbers PQ184803 for FlyingTortilla and PQ184837 for ScarletRaider. This project has also been deposited in NCBI Sequence Read Archive databases (SRA) under accession numbers SRR31899099 for FlyingTortilla and SRR31899096 for ScarletRaider. The BioProject accession no. for both phages is PRJNA488469 .

## AFTER EPUB

[This article was published on 15 April 2025 with incorrect spelling of the author, Ashleigh Crawford. The spelling was corrected in the current version, posted on 25 April 2025.]
